# Dietary calcium supplementation promotes the accumulation of intramuscular fat

**DOI:** 10.1186/s40104-021-00619-6

**Published:** 2021-09-10

**Authors:** Zhiwang Zhang, Tingli Pan, Yu Sun, Siqi Liu, Ziyi Song, Haojie Zhang, Yixing Li, Lei Zhou

**Affiliations:** State Key Laboratory for Conservation and Utilization of Subtropical Agro-bioresources, College of Animal Science and Technology, Guangxi University, Guangxi Zhuang Autonomous Region, Nanning, 530004 People’s Republic of China

**Keywords:** C2C12, Calcium, Intramuscular fat, Meat quality, Pig

## Abstract

**Background:**

In the livestock industry, intramuscular fat content is a key factor affecting meat quality. Many studies have shown that dietary calcium supplementation is closely related to lipid metabolism. However, few studies have examined the relationship between dietary calcium supplementation and intramuscular fat accumulation.

**Methods:**

Here, we used C2C12 cells, C57BL/6 mice (*n* = 8) and three-way cross-breeding pigs (Duroc×Landrace×Large white) (*n* = 10) to study the effect of calcium addition on intramuscular fat accumulation. In vitro, we used calcium chloride to adjust the calcium levels in the medium (2 mmol/L or 3 mmol/L). Then we measured various indicators. *In vivo*, calcium carbonate was used to regulate calcium levels in feeds (Mice: 0.5% calcium or 1.2% calcium) (Pigs: 0.9% calcium or 1.5% calcium). Then we tested the mice gastrocnemius muscle triglyceride content, pig longissimus dorsi muscle meat quality and lipidomics.

**Results:**

*In vitro*, calcium addition (3 mmol/L) had no significant effect on cell proliferation, but promoted the differentiation of C2C12 cells into slow-twitch fibers. Calcium supplementation increased triglyceride accumulation in C2C12 cells. Calcium addition increased the number of mitochondria and also increased the calcium level in the mitochondria and reduced the of key enzymes activity involved in β-oxidation such as acyl-coenzyme A dehydrogenase. Decreasing mitochondrial calcium level can alleviate lipid accumulation induced by calcium addition. In addition, calcium addition also reduced the glycolytic capacity and glycolytic conversion rate of C2C12 cells. *In vivo*, dietary calcium supplementation (1.2%) promoted the accumulation of triglycerides in the gastrocnemius muscle of mice. Dietary calcium supplementation (1.5%) had no effect on pig weight, but significantly improved the flesh color of the longissimus dorsi muscle, reduced the backfat thickness and increased intramuscular fat content in pigs. Besides, calcium addition had no effect on longissimus dorsi pH, electrical conductivity and shear force.

**Conclusions:**

These results suggest that calcium addition promotes intramuscular fat accumulation by inhibiting the oxidation of fatty acids. These findings provide a new tool for increasing intramuscular fat content and an economical strategy for improving meat quality.

## Introduction

In the meat production industry, subcutaneous and visceral fat pads are considered to be useless [1, 2]. However, intramuscular fat (IMF) is one of the criteria used to evaluate meat quality [3]. IMF is closely related to meat quality, affecting characteristics such as flavor, water retention and tenderness [4] and high IMF content is believed to promote sensory experience during eating [5]. Besides, IMF can positively improves characteristics such as the microstructure of meat, retain higher water content and provide physical protection against muscle dehydration [6]. Although high IMF is beneficial to meat quality, reducing carcass fat content is the current direction of animal husbandry production [7]. Reducing carcass fat content while increasing IMF has become a struggle in current research [7, 8].

Many studies have shown that dietary calcium is closely related to fat accumulation [9, 10]. High extracellular calcium attenuates adipogenesis in 3T3-L1 preadipocytes [11]. Sun et al. demonstrated that high calcium can relieve obesity induced by a high-fat diet in mice [12]. High calcium (1.2%) and vitamin D (ten times higher than the recommended level of 1,000 IU/kg) intake can activate the calcium-mediated apoptotic pathway in adipose tissue. Targeting this pathway with vitamin D and calcium supplementation could contribute to the prevention or treatment of obesity [13]. There is also a potential relationship between calcium and IMF. A reduction in dietary protein promotes the accumulation of IMF in pigs and also changes the calcium signal [8]. Unfortunately, there have been few studies on the effect of dietary calcium supplementation on IMF in the livestock industry. However, studies have shown that the addition of dietary vitamin D can improve the color of the meat [14], and the injection of CaCl_2_ solution after slaughter can enhance the color of the meat [15]. These studies suggest that calcium content may affect the quality of meat.

The relationship between calcium and fat metabolism is very close, so we assume that dietary calcium levels have an impact on the accumulation of IMF. Starting with an exploration to improve the quality of meat products, our research studied the effect of calcium addition on lipid accumulation in muscle cells and the effects of dietary calcium supplementation on the IMF content of mice and pigs. In addition, we also analyzed the signaling pathways that affect IMF through lipidomics. This research may provide a direction for reducing carcass fat content and increase IMF content to improve meat quality.

## Methods

### Animals and diets

#### Mice and sample collection

Eight week old C57BL/6 J male mice (purchased from the Animal Experiment Center of Guangxi Medical University, Nanning, China) were divided into two groups (*n* = 8): CN0.5, normal calcium group (0.5%); and CN1.2, calcium supplementation group (1.2%). The mice were housed in cages in a room with a temperature of 22 °C ± 2 °C, and a 12 h dark/12 h bright schedule with free access to water and food. The reference standard for feed formula in the control group was the American Institute of Nutrition (AIN93) standard. The ingredient composition of mice feed is in Table 1. The calcium content in American Institute of Nutrition (AIN93) standard mice feed is 0.5%. After referring to other groups and our previous research, we chose 1.2% calcium in our calcium supplementary feed [16, 17]. Calcium carbonate was used to regulate calcium content in the calcium supplementation group.

**Table 1:** Ingredient composition of mice feed

Ingredients, g/kg	CN0.5 group	CN1.2 group
**Cornstarch**	397	394.2
**Casein ( ≥85% protein )**	200	198.6
**Dextrinized cornstarch**	132	131.1
**Sucrose**	100	99.3
**Soybean oil (no additives)**	70	69.5
**Fiber**	50	49.7
**Calcium**	5	12
**Other mineral mix**	30	29.8
**Vitamin mix**	10	9.9
**L-Cystine**	3	2.98
**Choline bitartrate**	2.5	2.48
**Tert-butyhydroquinone**	0.014	0.0139

After 10 weeks of feeding, all mice were fasted for 4 h, and the mice were anesthetized with ether, and then euthanized by cervical dislocation. The blood glucose level was measured immediately by blood glucose meter using the matched blood glucose test paper (Finetest, Infopia Co.,Ltd., Korea) and the gastrocnemius muscle of the mice was placed in a 1.5 mL centrifuge tube, then immediately freezed in liquid nitrogen and stored at − 80 °C.

#### Pigs and sample collection

Duroc × Landrace × Large white pigs (120 days old) castrated boars were divided into two groups (*n* = 10): NC, normal calcium group; and HC, calcium supplementation group. The normal pig feed was tested by the Analysis and Testing Center of Guangxi Zhuang Autonomous Region (Nanning, China) and the calcium concentration was found to be 0.9% (Table 2). We used calcium carbonate to adjust the calcium content of the feed in the HC group. The adjusted calcium concentration was 1.5% (Table 2). The pigs were raised in the Jiaxing pig farm in Nanning, with natural ventilation and the temperature was about 20–25 °C. The pigs were given in three daily meals. Weight of pigs was measured and recorded individually at the beginning of the experiment and before slaughter.

**Table 2:** Nutritional composition of pig feed

Nutrient, %	NC group	HC group
**Moistore**	27.5	27.4
**Crude protein**	44.2	44
**Crude fat**	9.3	9.2
**Crude ash**	8.4	8.3
**Crude fiber**	8.51	8.4
**Total phosphorus**	1.19	1.2
**Calcium**	0.9	1.5

*NC* normal calcium diet, *HC* high calcium diet.

After 60 days of feeding, pigs were slaughtered in two consecutive days. The pigs were fasted for 12 h. Then pigs were euthanized by electric stunning and exsanguination. Using vernier calipers to measure pig’s backfat thickness. Then take the longissimus dorsi muscle into multiple parts and pack them on ice for testing pH, conductivity, flesh color, and shear force. In addition, the longissimus dorsi muscle was frozen in liquid nitrogen and store at − 80 °C for lipidomics.

The protocol of mice and pigs experiments were approved by The Committee on Animal Experiments of Guangxi University (GXU2020–287) and were in compliance with relevant ethical regulations.

### Cell culture and differentiation

C2C12 cells were kept in the laboratory and cultured in Dulbecco’s Modified Eagle Medium (DMEM) (1.8 mmol/L calcium, Gibco, Beijing, China) containing 10% (v/v) Fetal Bovine Serum (FBS) and 1% (v/v) antibiotic in a humidified incubator at 5% CO_2_ at 37 °C. CaCl_2_ was used to adjust the calcium content in the medium. We used 2 mmol/L calcium concentration as the control group and 3 mmol/L calcium concentration as the calcium supplementation group. Cells were differentiated by incubation with 2% horse serum for 7 d [18]. After the differentiation is complete, Myoglobin (Myog) and Myosin heavy chain (Myhc) are tested as a marker gene for differentiation to determine whether the cells are differentiated. Cells were treated with oleic acid (OA) / palmitic acid (PA) for 24 h to induce lipid accumulation after the incubation.

### Cell viability assay

Cell viability was tested with a commercially available kit (Cell Counting Kit, 40203ES60, Yeasen, Shanghai, China). C2C12 cells were seeded in 100 μL of DMEM in a 96-well plate and placed in a 5% CO_2_ incubator at 37 °C for 24 h. Then, CaCl_2_ was added to adjust the calcium concentration. After 12 h, 10 μL CCK-8 solution was added to the wells. After 2 h, the absorbance at 450 nm was measured with a microplate reader.

### Real-time quantitative polymerase chain reaction(qPCR)

Total RNA was extracted by RNA Kit (GenStar, Stock number: P111–01) from C2C12 cells, and 1 μg RNA was used as a template to reverse transcribe to cDNA. Reverse transcription conditions were 72 °C, 10 min; 42 °C, 60 min; 72 °C, 10 min; 12 °C, 5 min. The qPCR was performed after reverse transcription was completed. The reaction mixture contained 2 μL of primers (forward and reverse primers, 1 μL each), 1 μL of cDNA, 10 μL 2X Realstar Green Fast Mixture and 7 μL DEPC water. The reaction conditions were 94 °C, 2 min, 94 °C, 30 s, followed by 40 cycles of 56 °C, 30 s, and 72 °C, 2 min. The housekeeping gene used was β-actin. The primer sequence is shown in Table 3.

**Table 3:** qPCR primer sequence

Gene	Sequence(5'→3')
*MyOG*	F: AGGAAGTCTGTGTCGGTGGA
	R: AGGCGCTCAATGTACTGGAT
*MyHC*	F: GAATGGCAAGACGGTGACTGTG
	R: GGAAGCGTAGCGCTCCTTGAG
*MEF2C*	F: ACCAGGACAAGGAATGGGAG
	R: GGCGGCATGTTATGTAGGTG
*TNNI1*	F: ATGCCGGAAGTTGAGAGGAA
	R: CTGAAGGGCACTGAGAGACA
*PGC-1α*	F: AGCCTCTTTGCCCAGATCTT
	R: GGCAATCCGTCTTCATCCAC
*TNNC1*	F: GCAAGGTGATGAGGATGCTG
	R: GACTTCCCTTTGCTGTCGTC
*MYH1*	F: GGACCCACGGTCGAAGTTG
	R: CCCGAAAACGGCCATCT
*MYH4*	F: CAATCAGGAACCTTCGGAACAC
	R: GTCCTGGCCTCTGAGAGCAT
*TNNC2*	F: CGGCTCCATCGACTTTGAAG
	R: AGCAGCTCATCGATCTCCTC
*TNNI2*	F: GATGAGGAGAAGCGCAACAG
	R: TTTCTCCTCTTCAGCCACGT
*β-actin*	F: CAGCCTTCCTTCTTGGGTAT
	R: TGGCATAGAGGTCTTTACGG

*MyOG* Myogenin, *MyHC* Myosin Heavy Chain, *MEF2C* Myocyte Enhancer Factor 2C, *TNNI1* Troponin I1 Slow Skeletal Type, *PGC-1α* PPARG Coactivator 1 alpha, *TNNC1* Troponin C1 Slow Skeletal and Cardiac Type, *MYH1* Myosin Heavy Chain 1, *MYH4* Myosin Heavy Chain 4, *TNNC2* Troponin C2 Fast Skeletal Type, *TNNI2* Troponin I2 Fast Skeletal Type.

### Immunofluorescence

After C2C12 cell differentiation, the culture medium was discarded and washed once with PBS. Pre-cooled 4% paraformaldehyde (350 μL) was added to fix cells at room temperature for 30 min and then samples were washed twice with PBS. Next, 0.5% TritonX-100 (350 μL) was added to permeabilize the cells for 10 min at room temperature, followed by washing with PBS two times. Each well was blocked with 350 μL 5% BSA for 30 min at 37 °C, then washed twice with PBS. Primary antibody diluent (350 μL) was added to each well, incubated overnight at 4 °C and washed twice with PBS. Next, secondary antibody diluent (350 μL) was added and incubated in the dark for 1 h [19]. The Tecan infinite M200 PRO was used to measure absorbance at Ex/Em = 495/560 nm, and images were collected with a fluorescence microscope.

### Cell scratch assay

C2C12 cells were seeded in a six-well plate and placed in a 37 °C, 5% CO_2_ incubator for 12 h. When the cells evenly covered the bottom of the 6 well plate, a sterile pipette tip was used to draw a straight line in the well plate. Next, the healing degree of cell scratches at 0, 6, 12 and 24 h was observed and photographed with a microscope (Olympus, CKX41SF, Tokyo, Japan).

### Oil red O staining

C2C12 cell culture medium was removed from the 24-well cell culture plate and rinsed with PBS twice and fixed with 4% paraformaldehyde for 30 min. The cells were stained with oil red O (Solarbio, O8010, Beijing, China) for 30 min, rinsed with 60% isopropanol and rinsed three times with PBS [20]. Finally, samples were visualized using a 100X microscope (Olympus, CKX41SF, Tokyo, Japan) to observe and collect images. For the oil red O staining of mouse muscles, the mouse gastrocnemius muscle was first frozen sectioned. The slices were dipped into oil red dye solution for 8-10 min (covered to avoid light). Then the slices were took out, and stayed for 3 s and then immersed in 60% isopropanol 2 times, 3 s and 5 s respectively. The slices were immersed in pure water and soaked twice, each for 10 s. After that, we took out the slices, stayed for 3 s and then immersed in hematoxylin for 3-5 min. Then microscope inspection, image acquisition and analysis were performed.

### Cell/tissue TG assay

The tissue/cell triglyceride (TG) kit (Pulilai, Beijing, China) was used to determine triglyceride content. To lyse cells, RIPA lysis buffer (200 μL) containing 2 μL PMSF was added and samples were incubated in a shaker at 4 °C for 1 h. Samples were then transferred to a centrifuge tube and centrifuged at 4 °C and 12,000 r/min for 10 min. The supernatant was collected to detect triglyceride and protein content. For tissue processing, 1 mL of RIPA lysis buffer containing 10 μL PMSF was added to 100 mg tissue in a centrifuge tube. Steel balls were added to grind with a tissue disrupter (Tissue Lyser II) and the tissues were lysed overnight on a shaker at 4 °C. The lysate was centrifuged at 4 °C and 12,000 r/min for 10 min, and the supernatant was taken to detect triglyceride and protein content. TG/glycerin was normalized by protein levels.

### Calcium determination

The calcium concentrations of the cytoplasm, mitochondria and endoplasmic reticulum were measured using the calcium ion fluorescent probes, Fluo-4 AM, Rhod-2 AM and Fluo-5 N (Yeasen, Shanghai, China), respectively [21, 22]. Working solutions of Fluo-4 AM, Rhod-2 AM and Fluo-5 N were prepared according to the kit instructions. The treated cells were washed three times with an HBSS solution (without calcium). Working solution (300 μL) was added to each well and incubated at 37 °C for 30 min. Cells were washed two times with 0.5 mL HBSS containing 2 μL EGTA, followed by 1 wash with the HBSS solution. Finally 300 μL HBSS solution was added to each well, incubated at 37 °C for 15 min. The fluorescence levels of cytoplasm Fluo-4 AM and endoplasmic reticulum Fluo-5 N were determined at Ex/Em = 494/516 nm. The fluorescence of mitochondria Rhod-2 AM was measured at Ex/Em = 516/549 nm and normalized based on cell protein concentration.

### Quantification PCR of mitochondrial DNA

Use PCR lysis solution to extract cell DNA, and then perform fluorescence quantitative PCR to quantify nuclear DNA and mitochondrial DNA separately. The ratio of mitochondrial DNA to nuclear DNA represents the content of mitochondrial DNA [18].

### Enzyme activity detection

Glycogen synthase (GS), glycogen phosphorylase (GPa) and acyl-CoA dehydrogenase enzyme activity were tested using the enzyme activity detection kits (GPa test kit, BC3345, Solarbio, Beijing, China; GCS kit, BC3335, Solarbio; Acyl-CoA dehydrogenase kit, GMS50121.1.1, Genmed, Shanghai, China). Cell lysates were prepared according to the instructions of the corresponding kit, and the absorbance or fluorescence was measured with a multifunctional microplate reader (Tecan Infinite M200 PRO) at the corresponding wavelength.

### Western blot

C2C12 cells or mice gastrocnemius tissues were lysed with RIPA lysis buffer containing 1% PMSF. The total protein concentration was determined according to the instructions of the BCA protein assay kit (Beyotime, Shanghai, China). The protein sample and 4X loading buffer were mixed at a ratio of 3:1, heated at 100 °C for 10 min and SDS-PAGE was performed. After separation, the proteins were transferred to polyvinylidene fluoride (PVDF) membranes, and then blocked with TBST containing 5% skim milk powder for 1 h at room temperature. The membranes were incubated with the primary antibody overnight at 4 °C. The primary antibodies included anti-slow skeletal myosin heavy chain (1:1,000; ab11083, Abcam, Cambridge, UK), anti-fast skeletal myosin heavy chain (1:1,000; ab51236, Abcam), and β-tubulin antibody (1:1,000; 2146 s, Cell Signaling Technology, Inc., Shanghai, China). Total OXPHOS Rodent WB Antibody Cocktail (Abcam, Cambridge, UK; ab110413), Mitochondrial membrane protein antibody (Abcam ab110414), Porin (an outer membrane marker; Abcam ab14734), Cytochrome c (an intermembrane space marker; Abcam ab110325), Complex Va (an inner membrane marker; Abcam ab110273), Complex III Core 1 (Abcam ab110252) and Cyclophilin D (matrix space marker; Abcam ab110324). Next, the membranes were incubated with the secondary antibody for 1 h at room temperature. After adding ECL ultra-sensitive luminescent liquid, the Image Lab (Bio-Rad) was used to detect chemiluminescence signals. Densitometry analysis was quantified using Image J software [23].

### Oxygen consumption rate (OCR) and extracellular acidification rate (ECAR) assays

C2C12 cells were seeded into the Seahorse 24-well plate, and the cells were treated with DMEM medium containing OA/PA (200 μmol/L/100 μmol/L) for 24 h after differentiation. The cells were washed twice with the detection solution, and incubated in a 37 °C CO_2_-free incubator for 1 h. For oxygen consumption rate (OCR) assays, 1 μmol/L oligomycin, 1 mol/L Trifluoromethoxy carbonylcyanide phenylhydrazone (FCCP), and 0.5 μmol/L Rotenone/Antimycin A (Rot/AA) were added [24]. For extracellular acidification rate (ECAR) assays, 10 mol/L glucose, 1 μmol/L oligomycin, and 50 mol/L 2-deoxyglucose (2-DG) were added. OCR and ECAR were automatically recorded (Seahorse, XF-24, Bioscience, North Billerica, MA, U.S.A.) and calculated with the Seahorse XF24 software.

### Nuclear magnetic resonance (NMR) measurement

The lean mass and fat mass were determined by NMR (Niumag QMR23-060H-I, Suzhou, China). The instrument was preheated for 30 min. After calibration of the instrument, the live mice or pig longissimus dorsi muscle tissue (about 100 g) was fixed into the measuring cylinder and the weight of the mice or tissue was input, and the data, such as fat mass and lean mass were obtained [25].

### Meat quality determination

The longissimus dorsi were cut with a knife. The sample thickness were about 2-3 cm. The sample size were about 5 × 10 cm. The pork were wrapped in a clean ziplock bag and placed on ice. The pH of the muscle was measured at 1 h and 24 h using a pH meter (pH-STAR, Matthaus, Germany). The electrical conductivity of pork was measured using a ketone body muscle conductivity meter (LF-STAR, Matthaus). The flesh color of the longissimus dorsi muscle was measured using a flesh color tester (OPTO-STAR, Matthaus). To determine tenderness, the shear force was used as the evaluation index. The 1 cm diameter meat column of the longissimus dorsi were taken by a hollow sampler avoiding the tendons. The shear force of the longissimus dorsi muscle was measured using a shear force tester [26] (C-LM38, Tenovo, China). Meat quality test had measured 7 times on different parts of the same piece of meat. The highest and lowest values were removed, and the average was taken to represent.

### Lipomics

Six pigs were selected from 10 pigs in each group, a total of 12 pigs were selected for lipidomics. The longissimus muscle tissue of pigs extract was analyzed by Ultra Performance Liquid Chromatography (UPLC; Shim-pack UFLC Shimadzu CBM30A) and Tandem mass spectrometry (MS/MS, QTRAP®). Samples were thawed on ice, and then 50 mg of sample was homogenized in 1 mL (methanol/MTBE and internal standard mixture) with a steel ball. The steel ball was removed and the mixture was shaken for 2 min. Next, 500 μL of water was added and the mixture was swirled for 1 min, and then centrifuged at 12,000 r/min for 10 min at 4 °C. The supernatant (500 μL) was collected and concentrated. The powder was dissolved with 100 μL mobile phase B, then stored at − 80 °C. Finally, the dissolved solution was transferred to the sample bottle for LC-MS/MS analysis.

The chromatographic conditions were as follows: column, Thermo C30 (2.6 μm, 2.1 mm × 100 mm); solvent system, A: acetonitrile/water (60/40 V, 0.04% acetic acid, 5 mmol/L ammonium formate), B: acetonitrile/isopropanol (10/90 V, 0.04% acetic acid, 5 mmol/L ammonium formate); temperature, 45 °C; flow rate, 0.35 mL/min; injection volume: 2 μL.

Mass spectrometry conditions: LIT and triple quadrupole (QQQ) scans were acquired on a triple quadrupole-linear ion trap mass spectrometer (QTRAP), which was equipped with an ESI Turbo Ion-Spray interface, operating in positive and negative ion mode. The ion source temperature was 550 °C, the ion spray voltage (IS) was 5,500 V. A specific set of MRM transitions were monitored for each period according to the metabolites eluted within this period.

### Statistics

All data were presented as the means ± SD. Statistical analysis was performed using the unpaired two-tailed t-test. The differences were considered statistically significant if *P* < 0.05 (∗) or *P* < 0.01 (∗∗). The lipidomics statistical analysis refers to our previous lipidomics analysis [27]. The criteria for determining differential metabolites were: Fold change ≥2 or ≤ 0.5, and Variable Importance in Projection (VIP) value ≥1.

## Results

### Effect of calcium on the differentiation and proliferation of C2C12 cells

**Fig. 1:**
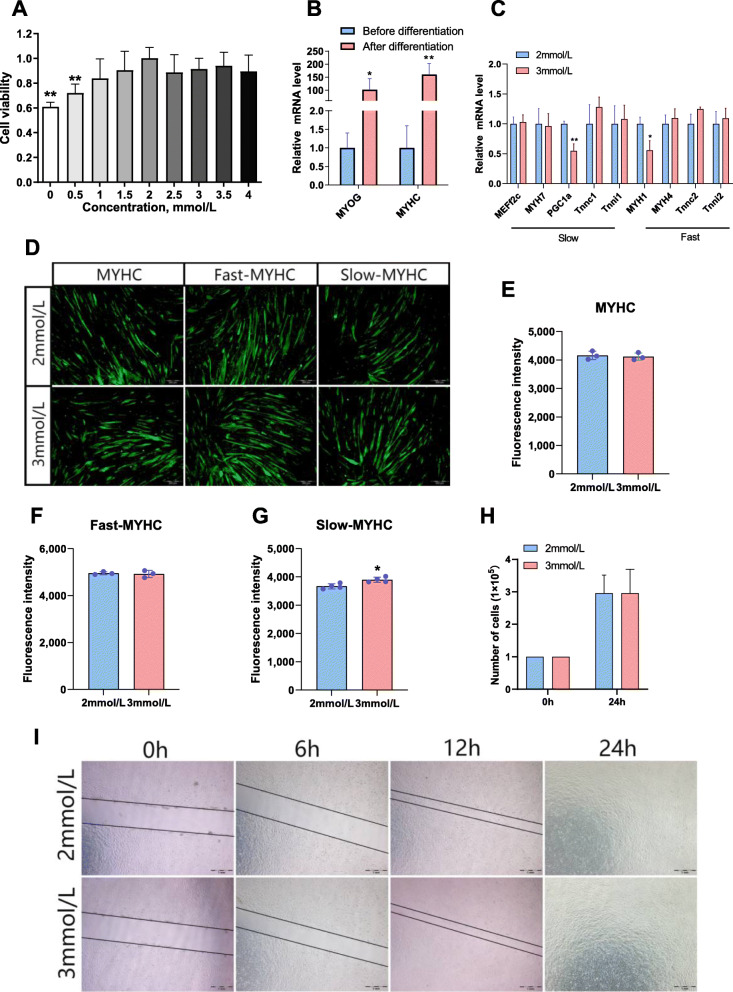
Effect of calcium on the differentiation and proliferation of C2C12 cells. **A** Cell counting assay (CCK-8 assay). **B** mRNA expression in C2C12 cells before and after differentiation. **C** mRNA expression of genes associated with regulation of slow-twitch fibers (*MEF2C, TNNI1, PGC-1α, MYH7, TNNC1*) and fast-twitch fibers (*MYH1, MYH4, TNNC2, TNNI2*), respectively. **D**, **E, F** and **G** Immunofluorescence analysis and quantification of myosin heavy chain (MYHC), slow-twitch myosin (Slow-MYHC) and fast-twitch myosin (Fast-MYHC) signals in C2C12 cells treated with different concentrations of calcium. **H** Effects of different concentrations of calcium on cell proliferation. **I** Wound-healing assay. 2 mmol/L, normal calcium level; 3 mmol/L, calcium supplementation. The data are expressed as the mean ± SD. *P < 0.05, **P < 0.01

In order to identify the appropriate calcium concentration to treat C2C12 cells, we first determined the effect of different concentrations of calcium on cell viability using the CCK-8 assay. It was found that 0 mol/L and 0.5 mmol/L calcium significantly inhibited the cell viability compared to the normal calcium concentration (2 mmol/L), while other concentrations did not significantly change cell viability (Fig. 1A) (0: *P* = 0.001; 0.5: *P* = 0.003). Based on the CCK-8 results, we chose 3 mmol/L as the concentration for calcium treatment. After 7 days of induction, we detected the mRNA expression levels of the differentiation marker genes *Myog* and *Myhc*. The results showed that the expression of the differentiation marker genes increased more than 100 fold, which proved that our differentiation was successful (Fig. 1B) (*Myog* *P* = 0.014; *Myhc* *P* = 0.003). We then studied the effect of calcium on cell differentiation by adding different concentrations of calcium while stimulating differentiation. We found that after the completion of differentiation, calcium supplementation reduced the mRNA expression of PPARG coactivator 1 alpha (*PGC1α*) and Myosin heavy chain 1 (*Myh1*) (Fig. 1C) (*PGC1α* *P* = 0.003; *Myh1* *P* = 0.01). Next, we performed immunofluorescence staining of muscle fibers (Fig. 1D), and found that calcium supplementation had no significant effect on the expression of *Myhc* (Fig. 1E) and fast-*Myhc* (Fig. 1F), but calcium supplementation significantly increased the expression of slow-Myhc (Fig. 1G) (*P* = 0.013). The cell proliferation results showed that calcium addition had no significant effect on the number of cells after 24 h treatment (Fig. 1H). The results of cell scratch assays demonstrated that calcium addition had no obvious effect on cell migration (Fig. 1I).

### Calcium supplementation increased the triglyceride content of differentiated C2C12 cells

**Fig. 2:**
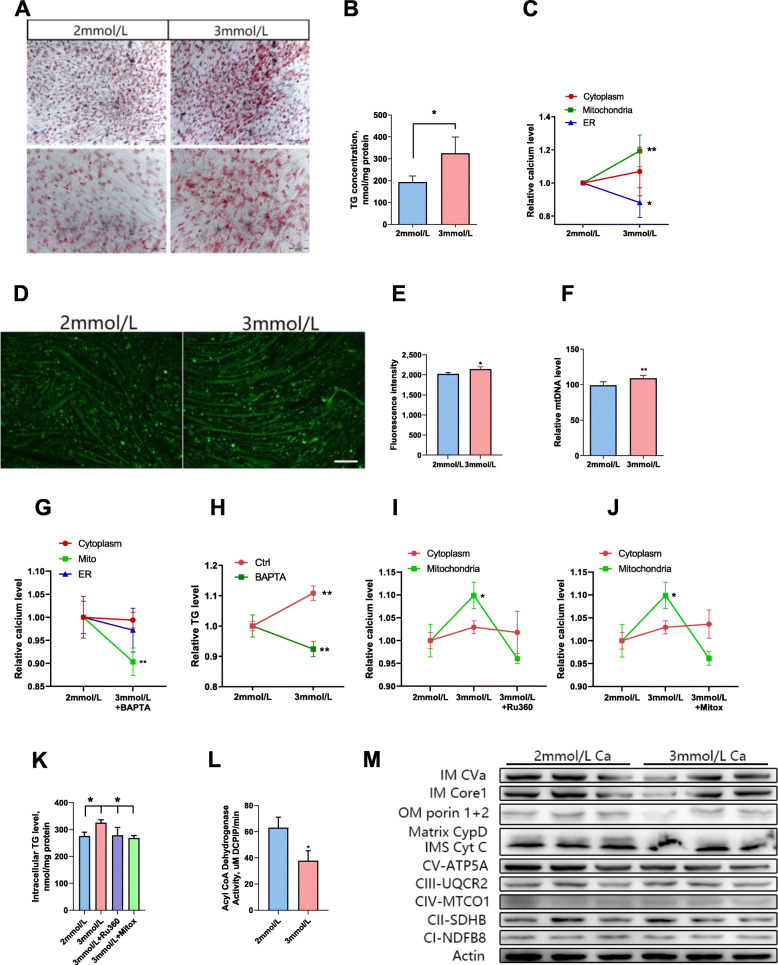
Calcium addition increases the triglyceride content of C2C12 cells. **A** Oil red O staining. **B** Triglyceride content. **C** Cytoplasm, mitochondria and endoplasmic reticulum calcium levels. **D** Mitochondrial staining. **E** Fluorescence intensity of mitochondrial staining. **F** Mitochondrial DNA quantification. **G** Changes of calcium levels in the cytoplasm, mitochondria and endoplasmic reticulum with or without chelation treatment (BAPTA, 0.1 μmol/L). **H** Intracellular triglyceride (TG) levels treated with different concentrations of calcium, with or without chelation. **I** Changes of calcium levels in the cytoplasm and mitochondria (10 nmol/L Ru360). **J** Changes of calcium levels in the cytoplasm and mitochondria (10 nmol/L Mitoxantrone). **K** Triglyceride content. **L** Acyl-CoA dehydrogenase activity. **M** Western blot of mitochondrial membrane proteins and redox proteins. The data are expressed as the mean ± SD. *P < 0.05, **P < 0.01

Oil red O staining (Fig. 2A) and triglyceride content (Fig. 2B) (*P* = 0.021) both showed that 3 mmol/L calcium significantly increased the triglyceride accumulation of differentiated C2C12 cells. Staining cells with specific dyes, we found that 3 mmol/L calcium significantly increased the calcium in the mitochondria and decreased the calcium in the endoplasmic reticulum (Fig. 2C). The mitochondria is the main location for energy metabolism in the cell [28]. Therefore, we performed mitochondrial staining on cells treated with different concentrations of calcium (Fig. 2D). Quantification of fluorescence intensity showed that 3 mmol/L calcium significantly increased the number of mitochondria (Fig. 2E). Quantitation of mitochondrial DNA also confirmed this conclusion (Fig. 2F). BAPTA treatment significantly reduced calcium levels in mitochondria (Fig. 2G). Subsequently, we tested the triglyceride content of the BAPTA-treated cells and found that chelating the calcium in the mitochondria significantly reduced the increase in triglyceride accumulation caused by 3 mmol/L calcium (Fig. 2H). Mitochondrial Calcium Uniporter (MCU) specific inhibitors Ru360 and Mitoxantrone (Mitox) can block MCU channel. The results showed that Ru360 and Mitox indeed reduced the calcium levels in mitochondria, but had no effect on the calcium in the cytoplasm (Fig. 2I & J). Ru360 and Mitox also reduced the triglyceride content caused by calcium addition (Fig. 2K) (*P* = 0.017). We assessed whether the increase in lipid accumulation was caused by impaired oxidation. An enzymatic activity measurement demonstrated that 3 mmol/L of calcium reduced the enzymatic activity of acyl-coenzyme A dehydrogenase (β oxidation limiting enzyme) (Fig. 2L). We also tested the expression of mitochondrial membrane and redox proteins, and found that the levels were similar to the in vivo results, and there was no significant change (Fig. 2M).

### The effect of calcium on glycolysis and the oxygen consumption rate

**Fig. 3:**
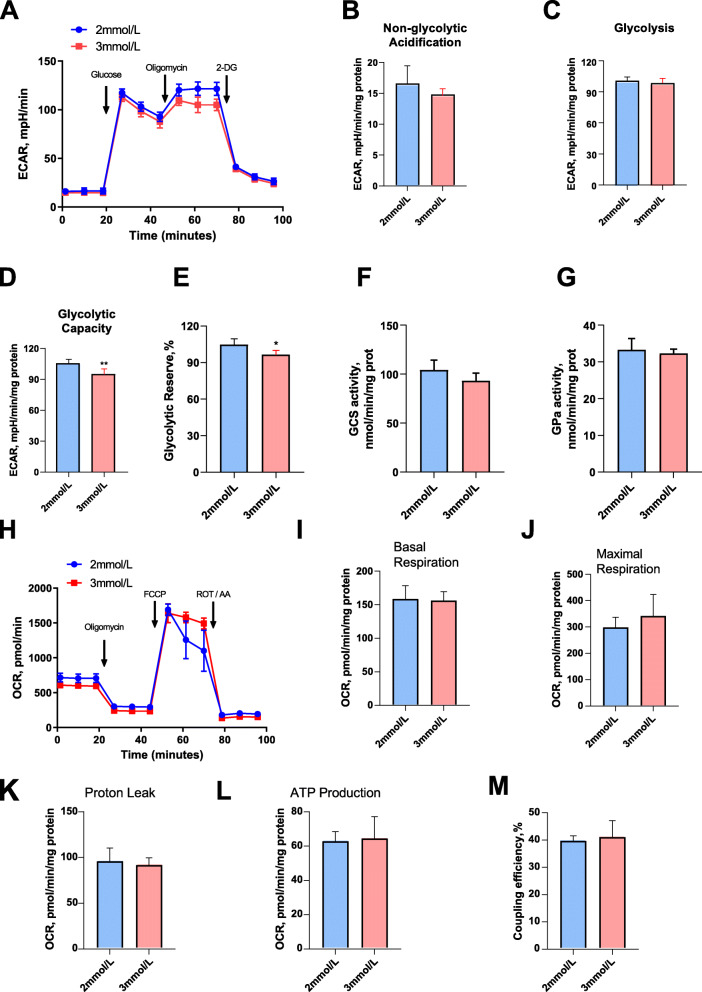
Effects of calcium supplementation on glycolysis and mitochondrial energy metabolism in C2C12 cells. **A** Extracellular acidification rate (ECAR) assay. **B** Non-glycolytic acidification. **C** Glycolytic. **D** Glycolytic capacity. **E** Glycolytic reserve. **F** Glycogen synthase activity. **G** Glycogen phosphorylase activity. **H** Oxygen consumption rate (OCR) assay. **I** Basal respiration. **J** Maximal respiration. **K** Proton leak respiration. **L** ATP production. **M** Coupling efficiency. The data are expressed as the mean ± SD. *P < 0.05, **P < 0.01

We assessed the effect of calcium on glycolysis (Fig. 3A). The quantitative results showed that calcium has no significant effect on non-glycolytic acidification (Fig. 3B) and glycolysis (Fig. 3C). However, calcium addition reduced the glycolytic capacity of C2C12 cells (Fig. 3D) (*P* = 0.009). Moreover, 3 mmol/L calcium also reduced the glycolytic conversion rate of cells (Fig. 3E) (*P* = 0.018). In order to further understand the effect of calcium on glucose metabolism, we tested the activities of glycogen synthase (Fig. 3F) and glycogen phosphorylase (Fig. 3G) and found that calcium had no significant effect on the activity of these two enzymes. We determined the effect of calcium on the oxygen consumption rate (Fig. 3H). The quantitative results showed that calcium had no significant effect on the basic oxygen consumption of cells (Fig. 3I) and maximum oxygen consumption rate (Fig. 3J). Calcium addition had no significant effect on the proton leak oxygen consumption (Fig. 3K), ATP production (Fig. 3L), or coupling efficiency (Fig. 3M) of C2C12 cells.

### Dietary calcium supplementation increased the accumulation of IMF in mice

**Fig. 4:**
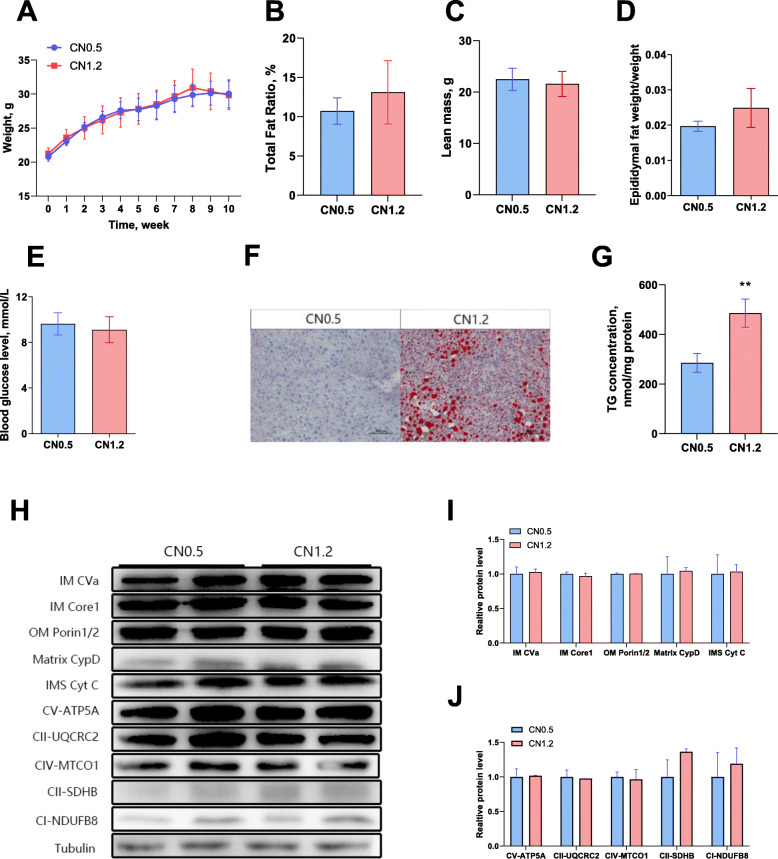
Effects of calcium supplementation diet on IMF in mice. **A** Mouse body weight in the normal calcium group (CN0.5) and calcium supplementation group (CN1.2). **B** Fat mass. **C** Lean mass. **D** Epididymal fat weight/body weight. **E** Blood glucose. **F** Gastrocnemius oil red O staining. **G** Gastrocnemius triglyceride content. **H I** & **J** Western blot and quantification of mitochondrial membrane proteins and redox related proteins. *n* = 8, The data are expressed as the mean ± SD. *P < 0.05, **P < 0.01

It was found that calcium-added feed had no significant effect on the weight of mice (Fig. 4A). NMR results showed that the total fat mass and lean mass of the mice did not significantly changed (Fig. 4B & C). Additionally, no significant changes in epididymal fat weight /body weight were observed (Fig. 4D). We tested the blood glucose levels of mice and found no significantly difference between the CN0.5 group and CN1.2 group (Fig. 4E). The oil red O and triglyceride content results (Fig. 4F & G) (G: *P* = 0.001) showed that dietary calcium addition significantly increased the IMF content of mice. We also determined the expression of mitochondrial membrane proteins (Fig. 4H & I) and redox-related proteins (Fig. 4H & J) in mouse gastrocnemius muscle and found that expression of mitochondrial membrane proteins and redox-related proteins did not significantly changed.

### Dietary calcium addition reduced the thickness of back fat and increased the content of IMF in pigs

**Fig. 5:**
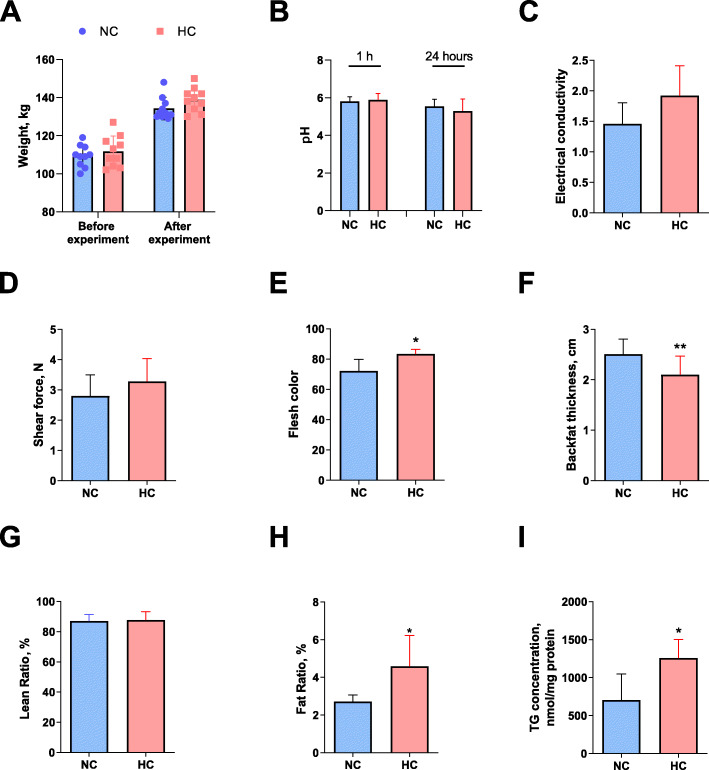
Effect of calcium supplementation on the pork quality of tri-crossbreeding pigs. **A** Pig weight. **B** Longissimus dorsi pH. **C** Electrical conductivity. **D** Shear force. **E** Flesh color. **F** Backfat thickness. **G** Lean ratio. **H** Fat ratio. **I** Triglyceride content. NC, normal calcium diet; HC, high calcium diet. *n* = 10, The data are expressed as the mean ± SD. *P < 0.05, **P < 0.01

There was no significant difference in the weight of pigs after two months of feeding (Fig. 5A). The longissimus dorsi muscle was taken to test the pH at 1 h and 24 h after slaughter, and no significant difference was found (Fig. 5B). After calcium supplementation in feed, the electrical conductivity of the longissimus dorsi muscle had an upward trend, but the difference was not significant (Fig. 5C). In addition, calcium addition in feed did not change its shear force (Fig. 5D). After testing the flesh color of the muscles, it was found that calcium addition feeding significantly increased the flesh color by about 15% (Fig. 5E) (*P* = 0.011). High-calcium feeding significantly reduced the backfat thickness (Fig. 5F) (*P* = 0.009). The NMR results showed that high-calcium feeding had no effect on the lean ratio (Fig. 5G) of muscle but significantly increased its fat content (means IMF content) (Fig. 5H) (*P* = 0.043). The triglyceride content results demonstrated that calcium supplementation significantly increased the triglyceride content of the longissimus dorsi muscle (Fig. 5I) (*P* = 0.039).

### Calcium addition affected pig IMF synthesis through the glycerolipid metabolism pathway

**Fig. 6:**
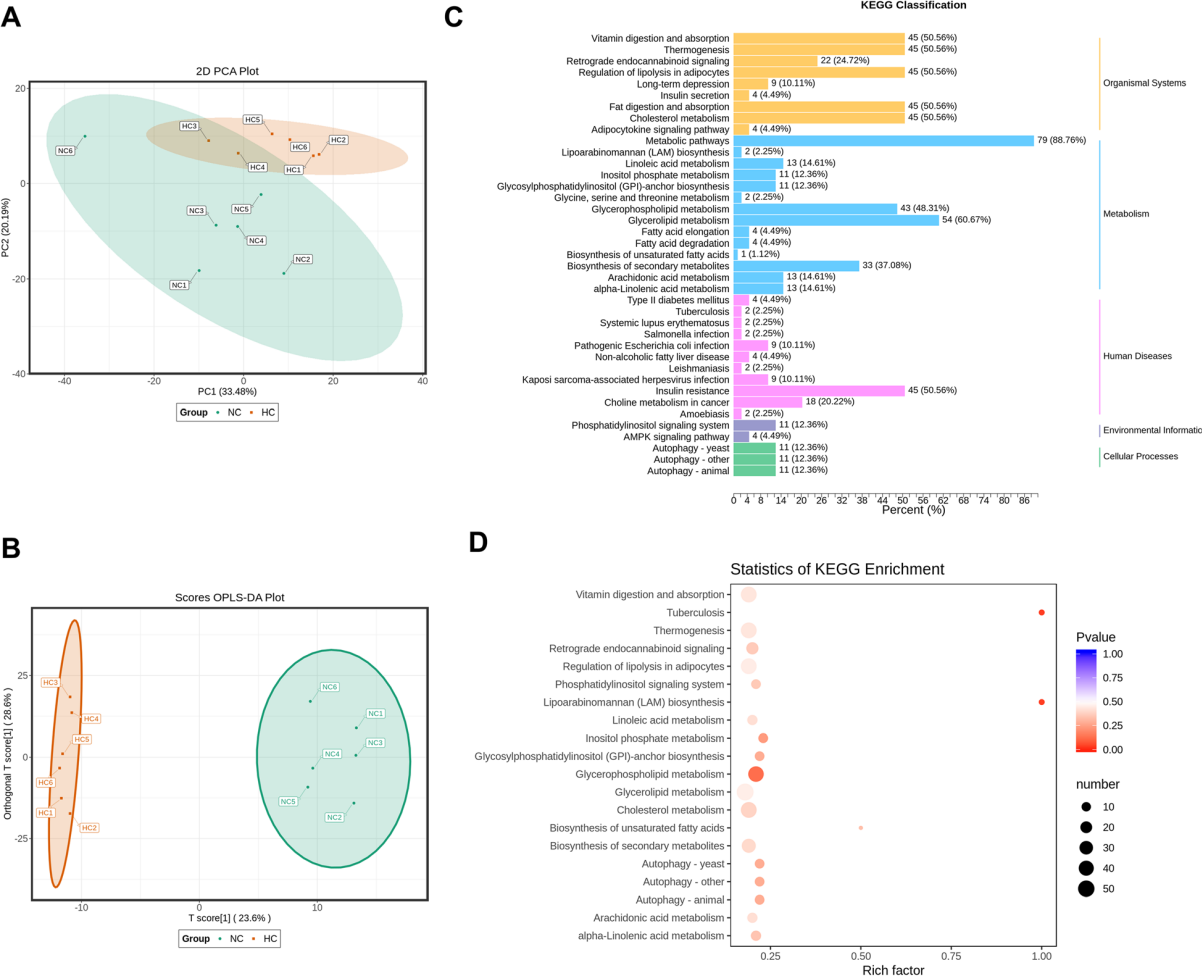
Pathways enriched by differential metabolites and RNA sequence. **A** Principal Component Analysis (PCA), and **B** Orthogonal Partial Least Squares Discriminant Analysis (OPLS-DA) of muscle tissue (*n* = 6). **C** KEGG classification enrichment of differential metabolites. **D** Statistics of KEGG enrichment. The larger the value, the greater the degree of enrichment. The closer the *P* value is to 0, the more significant the enrichment. The size of the dot in the figure represents the number of significantly different metabolites enriched in the corresponding pathway. NC, normal calcium diet; HC, high calcium diet

Using the UPLC-MS/MS platform, we detected 605 lipids from 12 pigs (6 pigs per group). Principal component analysis (PCA) revealed a grouping of the normal calcium group (NC) and calcium addition group (HC) in the first two components that accounted for 53.67% (PC1 33.48% & PC2 20.19%) of the variation (Fig. 6A). Orthogonal partial least squares discriminant analysis (OPLS-DA) distinguished the NC and HC groups well (Fig. 6B). We used the KEGG database [29] to annotate 100 differential metabolites and classify them (Fig. 6C). There were 79 differential metabolites (88.76%) enriched in the metabolic pathway, and 54 differential metabolites (60.67%) enriched in the glycerolipid metabolism pathway. After the enrichment analysis of the differential lipid metabolites by the KEGG pathway, we found that the highest enrichment was also the glycerolipid metabolism pathway (Fig. 6D).

## Discussion

This study determined the effect of calcium supplementation on lipid metabolism in C2C12 cells, mice and pigs. Our data showed that in vitro calcium addition promoted the formation of slow muscle fibers, increased the level of calcium in mitochondria, reduced the activity of β-oxidation key enzymes, and reduced the glycolytic capacity of muscle cells. In vivo, dietary calcium supplementation promoted the accumulation of IMF in mice, reduced the thickness of back fat, and increased IMF content in pigs.

Calcium regulation of mitochondrial activity is essential for high-energy-demanding excitable cells [30]. Skeletal muscles need to cope with a large range of activities, which means that they have great demands for energy [28]. Our study found that calcium supplementation in the culture medium increased the calcium level in the mitochondria of C2C12 cells (Fig. 2C). The mitochondria is the main site of energy metabolism. The tricarboxylic acid cycle and oxidative phosphorylation are mainly carried out in the mitochondria and cytoplasm [28, 30]. However, after testing the oxygen consumption rate of the cells, we found that calcium addition did not significantly affect oxidative phosphorylation (Fig. 3H-M). Acyl-coA synthetases catalyze the binding of fatty acids to coenzyme A to form fatty acyl-coA thioesters, which control the first step in the intracellular metabolism of fatty acids [13]. After detecting the activity of acyl-coA dehydrogenase, we found that calcium addition significantly reduced enzyme activity (Fig. 2L). This means that calcium may promote the accumulation of triglycerides by inhibiting the beta oxidation of fatty acids.

Meat is usually divided into red meat and white meat according to its color. Flesh color is one of the most important quality characteristics for consumers when purchasing meat. For example, bright red is ideal and is seen as a sign of the freshness [31]. The color of fresh and processed meat depends on the concentration of myoglobin, the chemical and physical state, the attached ligands (such as O_2_, CO and NO), and to some extent the surface structure of the meat [31]. Calcium supplementation feeding increased the longissimus dorsi color of pigs (Fig. 5E). This may indicate that calcium addition can increase the amount of myoglobin in pork.

Muscle fiber type can significantly affect meat quality [32]. Slow type muscle fibers are oxidative type muscle fibers. Research by Ryu, Y. C et al. showed that the higher the proportion of slow type muscle fibers, the better the meat quality [33]. And our results showed that calcium addition can increase the proportion of slow-type muscle fibers in cells (Fig. 1G), which indicates that calcium addition may regulate meat quality.

Glycerolipid metabolism is closely related to lipid storage. Calcium is also related to glycerolipid metabolism. Xiphias et al. found that calcineurin B homologous protein 1 (CHP1) is a regulatory protein for glycerolipid metabolism. The loss of CHP1 severely reduces fatty acid incorporation and storage [34]. Research by Lindsey et al. showed that Ca^2+^-ATPase regulates the glycerolipid synthesis network through ceramide [35]. After detecting the lipids of the longissimus dorsi muscle of pigs, we found that the differential metabolites of the glycerolipid metabolism pathway were abundantly enriched, which indicates that calcium promotes the accumulation of IMF by regulating glycerolipid metabolism.

## Conclusions

Our research confirms that calcium addition can increase the accumulation of IMF, which provides a feasible direction for the improvement of animal husbandry products. In addition, calcium addition also increases the flesh color of the longissimus dorsi muscle and reduces the backfat thickness. This may provide a direction for improving meat quality.

## Data Availability

The datasets used and/or analysed during the current study are available from the corresponding author on reasonable request.

## Abbreviations

IMF: Intramuscular fat
NMR: Nuclear magnetic resonance
OCR: Oxygen consumption rate
ECAR: Extracellular acidification rate
GS: Glycogen synthase
GPa: Glycogen phosphorylase
TG: Triglyceride
qPCR: Real-time quantitative polymerase chain reaction
Slow-Myhc: Slow skeletal myosin heavy chain
Fast-Myhc: Fast skeletal myosin heavy chain
PGC1α: PPARG coactivator 1 alpha
Myh1: Myosin heavy chain 1
MCU: Mitochondrial calcium uniporter
Mitox: Mitoxantrone
PCA: Principal component analysis
OPLS-DA: Orthogonal partial least squares discriminant analysis
